# Informing policy through evidence: A scoping review of factors that influence enrolment in community-based health insurance in East Africa

**DOI:** 10.1016/j.ghrp.2026.01.002

**Published:** 2026-02-03

**Authors:** Robert Lubajo, Sedona Sweeney, Olushayo Oluseun Olu

**Affiliations:** a World Health Organization Regional Office for Africa, Brazzaville, Congo; b London School of Hygiene and Tropical Medicine (LSHTM), London, UK; c The London School of Economics and Political Science (LSE), London, UK

**Keywords:** Community-based Health Insurance, Universal Health Coverage, Catastrophic Health Expenditure, Out-of-Pocket Payments, Socio-Ecological Model, East Africa

## Abstract

**Background:**

Community-based health insurance (CBHI) has emerged as a promising option to enhance the attainment of Universal Health Coverage in Low- and middle-income countries and Sub-Saharan Africa. However, CBHI schemes in Africa particularly in the eastern regions, grapple with chronic low enrolment, jeopardizing their sustainability and intended impact. Understanding the determinants of enrolment in these schemes is critical for designing effective strategies to boost participation. In this scoping review, we categorized and discussed these determinants across socio-ecological levels.

**Methods:**

We conducted a scoping review of literature using the Preferred Reporting Items for Systematic Reviews and Meta-Analysis Extended for Scoping Review checklist. Literature searches were conducted across academic databases to identify studies on determinants of enrolment to CBHI schemes in East Africa in June 2023 and updated in June 2025. Peer-reviewed, English-language studies using cross-sectional, case-control, qualitative, case studies, or mixed-methods designs were included which were conducted in East Africa and reported primary outcomes on determinants of CBHI enrolment (2000–2025). Excluded studies were reviews, non-peer reviewed articles, willingness to pay/willingness to enrol studies, and those on satisfaction, dropouts, or compulsory schemes (e.g., Rwanda post-2007, Seychelles as a high-income country). Data extraction and thematic analysis guided by a socio-ecological model framework were performed on the data.

**Results:**

A total of thirty articles met the inclusion criteria. The findings unveiled a broad spectrum of determinants influencing CBHI enrolment. At the individual level, key factors included awareness, socio-demographics, and personal predisposition. At the household level, household characteristics, social capital, and cohesion played significant roles. Community-level determinants included cultural beliefs, religion, and geographical location, while system-level factors were stakeholder influence, governance, benefit package design, premium structure, human resource management, supply chain, access to care, and referral systems.

**Conclusions:**

The findings emphasize the need for a holistic and multi-level approach to enhancing enrolment. Policymakers and stakeholders should integrate these determinants into interventions to strengthen CBHI schemes, expand healthcare access, and reinforce financial protection. Further studies are needed to explore the interplay between these factors.

## Background

Universal Health Coverage (UHC), which aims to guarantee that all individuals and communities have access to essential health services without incurring financial hardship, has emerged as a central priority in the global health agenda.^1^, ^2^ Rooted in equity and social justice, UHC is a flagship of the third Sustainable Development Goal (SDG 3) and the vision of the Alma Ata Declaration.^3^, ^4^, ^5^ Achieving UHC requires sustainable and equitable healthcare financing.^6^, ^7^, ^8^ Taxation and social health insurance (SHI) are two widely recognized health financing mechanisms and successfully implemented in many high-income countries.^9^, ^10^ Taxation pools funds through government revenue, while SHI relies on mandatory or voluntary contributions.^9^

However, in low- and middle-income countries (LMICs), both mechanisms (taxation and SHI) face significant challenges due to factors like low tax bases, large informal economies, limited government capacity, and high debt burdens.^11^, ^12^, ^13^ These constraints hinder the ability of governments to adequately fund health systems. A 2014 World Bank study highlighted that while LMICs carried approximately 90 % of the global disease burden, they accounted for just over 10 % of global health spending.^14^ As a result, individuals and households in LMICs bear a disproportionate share of healthcare costs, frequently encountering catastrophic health expenditure (CHE), where medical expenses exceed their ability to pay.^15^, ^16^, ^17^, ^18^ Regressive out-of-pocket (OOP) payments remain a primary mode of healthcare financing in many LMICs, depriving people of financial protection and slowing progress toward UHC.^19^ Each year, an estimated 44 million families (over 150 million people) experience CHE, and more than 25 million families (over 100 million people) are pushed into poverty due to healthcare-related expenses, and over 90 % of these cases occur in LMICs.^20^

Sub-Saharan Africa (SSA) faces even more acute challenges in financing healthcare. The region bears a particularly high disease burden, which further exacerbates existing financing gaps and strains fragile health systems.^21^, ^22^ Many SSA countries also contend with fragmented health financing structures, heavy dependence on external donor funding, and limited fiscal capacity to scale up prepayment and risk-pooling mechanisms like SHI.^23^, ^24^ These systemic constraints make it difficult to reduce OOP spending and expand financial protection for vulnerable populations. Addressing these issues requires context-specific strategies that take into account SSA's unique economic, demographic, and epidemiological characteristics to support progress toward achieving UHC.

Recognizing these challenges, CBHI has emerged as an alternative strategy to enhance coverage, especially for populations with limited access to formal employment or social protection.^25^, ^26^ CBHI refers to a non-profit health financing mechanism in which community members voluntarily contribute funds to covering the cost of health services. It primarily targets informal sector workers and rural populations who are often excluded from or lack access to formal health insurance schemes.^27^, ^28^ Typically managed at the local level, CBHI schemes emphasize social solidarity and risk-sharing, and they vary in structure, ranging from provider-based models to mutual health organizations and cooperative-based schemes.^29^ By pooling resources, CBHI enables cross-subsidization between the healthy and the sick, and between the rich and the poor, thereby reducing OOP spending and the risk of CHE while enhancing healthcare utilization.^30^, ^31^, ^32^, ^33^

Several countries in East Africa (EA) have actively promoted CBHI over the past two decades to improve healthcare access and financial protection.^21^, ^34^, ^35^, ^36^ However, enrolment in CBHI remains low, limiting its coverage, financial protection, and sustainability.^37^, ^38^ Despite CBHI’s potential, little is known about the factors influencing enrolment in EA, and no studies were identified that examined determinants of CBHI enrolment in EA, and none applied the socio-ecological model (SEM) framework. This gap limits context-specific understanding and underscores the need for research using theory-driven frameworks to explore enrolment factors within the region’s unique social and health system contexts. Understanding these context specific factors is critical for informing policy and programmatic interventions for improving financial protection and advancing UHC in EA. The SEM framework is particularly useful in this context as it guides the development of effective and more comprehensive interventions by explicitly considering multiple levels of influence incorporating behavioural, environmental and psychosocial contexts.^39^

Thus, this study aims to identify and categorize the key determinants influencing CBHI enrolment in EA using a SEM framework. By systematically reviewing existing literature and identifying determinants at individual, household, community, and system levels, this study contributes to addressing evidence gaps and will inform targeted strategies to enhance CBHI participation in EA and other similar settings.

## Methods

### Study design and setting

We conducted a scoping review of literature using the Arksey and O'Malley^40^ and Peters et al.^41^ frameworks. The Preferred Reporting Items for Systematic Reviews and Meta-Analysis Extension for Scoping Reviews (PRISMA-ScR) guideline was adopted for reporting the review process.^42^ A PRISMA-ScR flowchart was utilized in illustrating the selection of articles related to CBHI enrolment and its determinants. The protocol was not prospectively registered, as registration is not mandatory for scoping reviews. This aligns with PRISMA-ScR guidance, which encourages but does not require protocol registration.^42^

We developed a SEM framework informed by the studies of Sallis et al.^43^ and Daher,^44^ which conceptualizes determinants across four levels: individual, household, community and systems. This framework was used as an analytical lens to organize and interpret findings from the included studies, with determinants mapped across the four socio-ecological levels during data synthesis.

The study focused on EA, comprising 13 countries according to the African Development Bank (AfDB) classification.^45^ However, only 12 countries were included in the study based on study eligibility criteria with Seychelles being excluded from the study due to its classification as a high-income country according to the World Bank (WB),^45^ thus not aligning with the research focus on CBHI which is commonly practiced in LMICs. Rwanda was not excluded completely but studies in Rwanda after 2007 where CBHI became compulsory in the country^36^ were excluded because they are not informative about determinants of voluntary enrolment to CBHI.

### Data search

We conducted a systematic online database search on June 27, 2023 on the following databases: MEDLINE Ovid, EMBASE Ovid, SCOPUS, and Africa Index Medicus (AIM). The search strategy was complemented by additional approaches including snowballing technique and searching grey literature sources of Google Scholar and google, and websites of WHO, AfCDC and East African Health Research Commission (EAHRC). The search was updated by conducting a limited targeted supplementary literature search on all the above sources covering the period from original search cutoff to June 14, 2025 (date second search was conducted), using the same Boolean search strategies. The search strategy combined three main concepts: "**Enrolment**," "**Community-Based Health Insurance**," and "**East Africa**." A comprehensive search strategy was developed, incorporating synonyms and controlled vocabulary for each concept. There was no language restriction applied during the initial search. Grey literature search (google scholar and google) was performed until the 15th page (as relevance declines beyond this point) using the main search concepts above. The detailed search strategies for all databases are provided in supplementary material 1.

### Eligibility criteria

The criteria for inclusion were studies that were cross-sectional, case-control, qualitative, case studies, and mixed methods with primary outcome reporting on determinants of enrolment to CBHI schemes. Furthermore, studies conducted in EA,^45^ published in English, and peer-reviewed were included with the publication period spanning from January 1, 2000, to June 14, 2025. Conversely, systematic/scoping reviews, editorials, conference abstracts, policy documents, non-peer-reviewed documents, studies on willingness to pay (WTP) or willingness to enrol (WTE), those focused on satisfaction, dropouts or retention and those conducted outside EA, in high-income countries or when enrolment to CBHI is compulsory were excluded. As such, studies in Rwanda after 2007 when CBHI became compulsory^36^ were excluded because they are not informative about determinants of voluntary enrolment to CBHI given that enrolment was compulsory in the country. Seychelles was also excluded from the study due to its classification as a high-income country according to the WB, thus not aligning with the research focus on CBHI which is commonly practiced in LMICs.^45^

### Screening and data management

For the initial search (June 27, 2023), we used Mendeley reference manager^46^ to manage the records extracted from the above databases and other sources. Title and abstract screening were conducted independently by two reviewers (RL and SS) using Mendeley’s shared library feature. Search results from all databases and grey literature were imported into a private Mendeley group by RL, who also identified and removed the duplicates. The reviewers applied the inclusion and exclusion criteria manually and used tags and notes to record screening decisions. Articles marked for inclusion by either reviewer after screening the title and abstract proceeded to full text review and were placed on a separate folder on Mendeley. Discrepancies at this stage were resolved through discussion and consensus. Finally, the same reviewers (RL and SS) read the full text of the articles in the second folder to determine whether they fully satisfy the inclusion criteria, and any discrepancies were again resolved as indicated above. After this stage, RL saved all eligible articles in a new folder on Mendeley and enrolled them into the study.

On the second search conducted on June 12, 2025, the search records were exported to Rayyan, an online tool for systematic reviews^47^ by Author RL who also did the deduplication on Rayyan. Authors RL and OOO screened the titles and abstracts of the records blindly and resolved any conflicting decisions at this stage through discussion and consensus via teams and WhatsApp calls. The same authors (RL and OOO) did the full article screening of the selected articles against the selection and rejection criteria and any conflicting decisions were resolved as indicated above.

After all the articles were selected and enrolled for the study, author RL used a standard Microsoft Word based form to obtain data about the characteristics of the selected studies (see summary of the included studies' characteristics in Table 1) and authors SS and OOO verified the extracted data. The variables included in the form were the article’s number, author’s details, title of the study, location, study design, sample size and the outcome being measured.

**Table 1:** Summary of the included studies’ characteristics.

S/N	Study	Title of the study	Location	Study Design	Sample size	Outcome
1	Abdilwohab et al.^50^	Factors affecting enrolment status of households for community-based health insurance in a resource-limited peripheral area	Ethiopia	Mixed Methods	842 HHs	Enrolment
2	Atafu et al.^51^	Adverse selection and supply-side factors in the enrolment in community-based health insurance	Ethiopia	Mixed Methods	2008 HHs, 8 FGDs	Enrolment
3	Basaza et al.^52^	Community health insurance in Uganda: Why does enrolment remain low? A view from beneath	Uganda	Mixed methods	30 FGDs, 185 IDLs	Enrolment
4	Basaza et al.^53^	Low enrolment in Ugandan Community Health Insurance Schemes: underlying causes and policy implications	Uganda	Case Study	62 KIIs	Enrolment
5	Chanie et al.^54^	Determinants of enrolment in community-based health insurance among Households	Ethiopia	Case-control	268 HHs	Enrolment
6	Demissie et al.^55^	Barriers and Facilitators of Community-Based Health Insurance Membership in Rural Amhara Region	Ethiopia	Qualitative	6 FGDs	Membership
7	Fite et al*.*^56^	Factors associated with enrolment for community-based health insurance scheme	Ethiopia	Case-control	332 HHs	Enrolment
8	Kagaigai et al.^57^	Do household perceptions influence enrolment decisions into community-based health insurance schemes in Tanzania?	Tanzania	Cross-sectional	722 HHs	Enrolment
9	Kapologwe et al.^58^	Barriers and facilitators to enrolment and re-enrolment into the community health funds/Tiba Kwa Kadi (CHF/TIKA) in Tanzania: a cross-sectional inquiry on the effects of socio-demographic factors and social marketing strategies	Tanzania	Cross-sectional	466 HHs	Enrolment and renewal
10	Macha et al.^59^	Determinants of community health fund membership in Tanzania	Tanzania	Mix-methods	1225 HHs, 7959 IDLs	Membership
11	Mebratie et al*.*^60^	Enrolment in Ethiopia’s Community-Based Health Insurance Scheme	Ethiopia	Mixed methods	1224 HHs	Enrolment
12	Mirach et al.^61^	Determinants of community-based health insurance implementation in west Gojjam zone: a community based cross-sectional study	Ethiopia	Cross-sectional	648 IDLs	Implementation
13	Modest et al.^62^	Enrolment Status and Determinants of Improved Community Health Fund Among Households in Dodoma Tanzania	Tanzania	Cross sectional	424 IDLs	Enrolment
14	Mollel et al.^63^	Determinants of Household’s Membership to Community Health Fund Scheme in Central Tanzania: A Case of Mkalama District	Tanzania	Cross sectional	130 HHs	Membership
15	Moyehodie et al.^64^	The effects of individual and community-level factors on community-based health insurance enrolment of households in Ethiopia	Ethiopia	Cross-sectional	8663 IDLs	Enrolment
16	Mussa et al.^65^	Impact of conditional cash transfers on enrolment in community-based health insurance among female-headed households in south Gondar zone, Amhara region.	Ethiopia	Cross-sectional	365 HHs	Enrolment
17	Nageso et al.^66^	Enrolment in community-based health insurance program and the associated factors among households in Boricha district, Sidama Zone.	Ethiopia	Cross-sectional	646 HHs	Enrolment
18	Ngowi et al.^67^	Determinants of enrolment in the Improved Community Health Fund among household members.	Tanzania	Cross-sectional	403 IDLs	Enrolment
19	Nshakira-Rukundo et al*.*^68^	Determinants of Enrolment and Renewing of Community-Based Health Insurance in Households with Under-5 Children in Rural South-Western Uganda	Uganda	Cross-sectional	464 HHs	Enrolment and renewal
20	Taddesse et al.^69^	Determinants of enrolment decision in the community-based health insurance, Northwest Ethiopia: a case-control study	Ethiopia	Case control	148 IDLs	Enrolment
21	Kamau et al.^70^	Community Based Health Insurance Schemes: Lessons from Rural Kenya	Kenya	cross- sectional	285 IDLs	Enrolment
22	Kamuzora et al.^71^	Factors influencing implementation of the Community Health Fund in Tanzania	Tanzania	Qualitative	13 FGDs	Enrolment
23	Desalegn et al.^72^	Determinants of enrolment in community-based health insurance program among households in East Wollega Zone, west Ethiopia: Unmatched case-control study	Ethiopia	Case control	428 HHs	Enrolment
24	Elmi et al.^73^	Determinants of enrolment for community-based health insurance in Somali region of Ethiopia	Ethiopia	Case control	214 IDLs	Enrolment
25	Kassie et al.^74^	Predictors of community-based health insurance enrolment among reproductive-age women in Ethiopia based on the EDHS 2019 dataset: a study using SHAP analysis technique, 2024	Ethiopia	Cross-sectional	9013 IDLs	Enrolment
26	Handebo et al.^75^	Enrolment of reproductive age women in community-based health insurance: Evidence from 2019 Mini Ethiopian Demographic and Health Survey	Ethiopia	Cross-sectional	8885 IDLs	Enrolment
27	Demsash.^76^	Spatial distribution and geographical heterogeneity factors associated with households’ enrolment level in community-based health insurance	Ethiopia	Cross-sectional	8663 IDLs	Enrolment
28	Wodessa et al*.*^77^	Determinants of the decision to enrol in community-based health insurance among households in the West Guji Zone, Oromia State, southern Ethiopia, in 2022	Ethiopia	Case control	690 HHs	Enrolment
29	Debessa et al.^78^	Women’s enrolment in community-based health insurance and its determinants in Sidama national regional state, Ethiopia, 2024: A multi-level analysis	Ethiopia	Cross-sectional	845 HHs	Enrolment
30	Kebede.^79^	Exploring Factors Influencing Family’s Enrolment in Community-Based Health Insurance in the City of Gondar Peri-Urban Community, Northwest Ethiopia: A Health Belief Model Approach	Ethiopia	Cross-sectional	358 HHs	Enrolment

### Analysis and synthesis of data

We employed a narrative synthesis approach using both themes and a SEM framework to analyse the data due to the heterogeneity of the study design, setting and the result being measured.^48^, ^49^ We used thematic and framework analyses methods to identify emerging themes and then organized the findings within the SEM framework. We reviewed the literature on CBHI schemes to develop early themes to code the findings in accordance with the levels of the SEM framework. We then screened the results sections of all the eligible studies, with findings coded under established headings but with flexibility to include new themes. We updated the themes as the data analyses process proceeded. Similarly, we summarized the relevant findings from each study into a Microsoft word table (supplementary material 2).

## Results

### Outcome of literature search

**Fig. 1:**
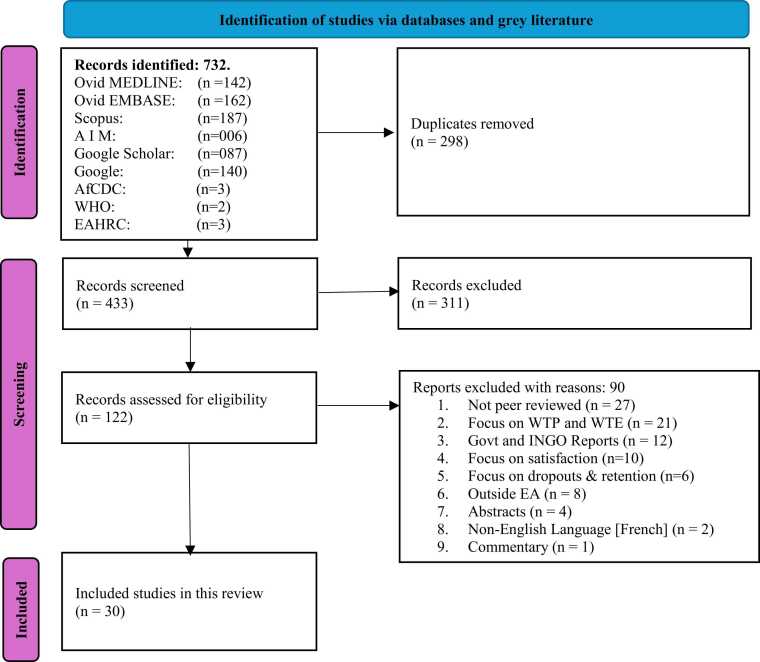
PRISMA-ScR flowchart.

The initial literature search, conducted on June 27, 2023, yielded 581 initial results. After removing 225 duplicate records, the remaining 356 records underwent a thorough evaluation based on heading, abstract, and full text for eligibility. Among these, 267 records were excluded based on abstracts and titles. The remaining 89 articles were assessed against the eligibility criteria, and ultimately, 22 articles were deemed suitable and included in the study. The second literature search conducted on June 14, 2025, yielded 151 articles. After removing 74 duplicates, the remaining 77 records underwent a thorough evaluation. Of these, 44 were eliminated based on titles and abstracts. The remaining 33 were assessed against the eligibility criteria and 8 more articles were deemed eligible and enrolled into the study. The entire search and selection process from both rounds was consolidated and is presented in a single PRISMA-ScR flowchart (Fig. 1).

### Characteristics of the included studies

As shown in Table 1, among the thirty articles selected for this research, nineteen were conducted in Ethiopia,^50^, ^51^, ^54^, ^55^, ^56^, ^60^, ^61^, ^64^, ^65^, ^66^, ^69^, ^72^, ^73^, ^74^, ^75^, ^76^, ^77^, ^78^, ^79^ seven in Tanzania,^57^, ^58^, ^59^, ^62^, ^63^, ^67^, ^71^ three in Uganda^52^, ^53^, ^68^ and only one in Kenya.^70^ The selected studies got published between 2007 and 2025, with 53.3 % of the studies published in the past five years (2021–2025). The research methodologies varied, with sixteen studies (53 %) employing cross-sectional designs,^57^, ^58^, ^61^, ^62^, ^63^, ^64^, ^65^, ^66^, ^67^, ^68^, ^70^, ^74^, ^75^, ^76^, ^78^, ^79^ five (17 %) utilizing mixed methods,^50^, ^51^, ^53^, ^59^, ^60^ two (7 %) using qualitative approaches,^55^, ^71^ six (20 %) adopting case control designs^54^, ^56^, ^69^, ^72^, ^73^, ^77^ and one (3 %) conducting a case study.^53^

The studies involved the sampling of both households and individuals, with some also incorporating focused group discussions (FGDs). Specifically, 14 studies collected data from households,^50^, ^54^, ^56^, ^57^, ^58^, ^60^, ^63^, ^65^, ^66^, ^68^, ^72^, ^77^, ^78^, ^79^ ten gathered information from individuals,^61^, ^62^, ^64^, ^67^, ^69^, ^70^, ^73^, ^74^, ^75^, ^76^ four utilized data from focused group discussions or key informant interviews^52^, ^53^, ^55^, ^71^ and two collected data from both households and individuals.^51^, ^59^ The sample sizes varied across the studies, with the number of households interviewed ranging from 130 to 1224, while the number of individuals interviewed ranged from 214 to 9013.

Regarding the focus of the studies, 24 examined the determinants of enrolment into CBHI schemes,^50^, ^51^, ^52^, ^53^, ^54^, ^56^, ^57^, ^60^, ^62^, ^64^, ^65^, ^66^, ^67^, ^69^, ^70^, ^71^, ^72^, ^73^, ^74^, ^75^, ^76^, ^77^, ^78^, ^79^ two explored the determinants of both enrolment and renewal of CBHI,^58^, ^68^ three studied membership aspects^55^, ^59^, ^63^ and one study specifically examined the determinants of CBHI implementation.^61^ Further information on the characteristics of the included papers is summarized in supplementary material 2.

The included studies showed heterogeneity in geographical coverage, design, population, and sample size. Most were conducted in Ethiopia, followed by Tanzania, Uganda, and Kenya, with none from the other eight eligible EA countries. Cross-sectional study designs were predominant, followed by case-control, mixed-methods, qualitative, and case study. Study populations included households, individuals, FGDs and KIIs as summarised on Table 1.

### Commonly reported determinants of enrolment to CBHI schemes in EA by study design

**Fig. 2:**
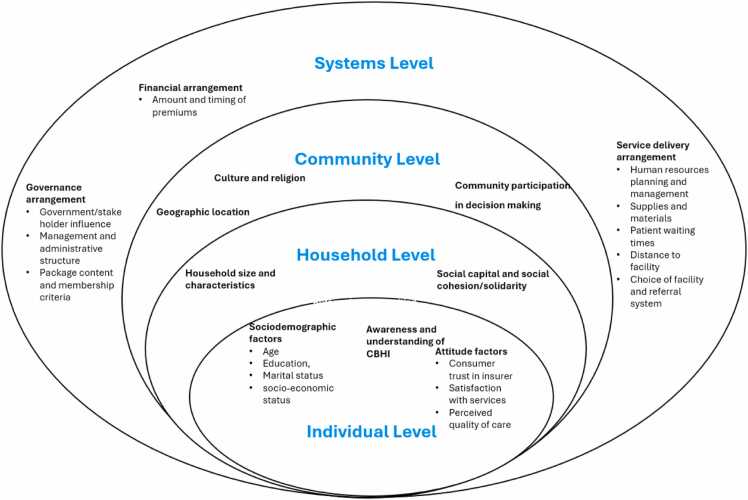
Populated SEM Framework.

Enrolment in CBHI schemes in EA is influenced by several factors. These findings, summarised by study design below, are synthesized visually in Fig. 2 (Populated SEM framework), illustrating how enablers and barriers to CBHI enrolment operate across individual, household, community and system levels. A structured narrative summary of these determinants is provided in Table 2 and a summary of results for each individual study is provided in supplementary material 2.

**Table 2:** Summary of the commonly reported determinants.

**Socio-ecological level**	**Themes**	**Sub-themes**	**Enablers**	**Barriers**
Individual level	Consumer awareness and understanding of CBHI principles	-	• Awareness and access to information about CBHI schemes.^51^, ^53^, ^56^, ^57^, ^63^, ^67^, ^68^, ^73^, ^76^	• Lack of awareness and access to accurate information about CBHI.^51^, ^52^, ^66^, ^72^, ^74^ • Lack of understanding about the insurance system/risk pooling.^51^, ^52^, ^57^, ^59^, ^70^, ^71^ • Increased media exposure was associated with a decreased likelihood of CBHI enrolment.^76^
	Attitude factors	Consumer trust in insurer	• Trust in the CBHI program.^53^, ^68^, ^77^, ^79^ • Active mobilization by scheme members.^53^, ^68^	• Distrust in scheme managers due to perceived lack of transparency & accountability, previous experience of fraud & corruption and low sense of ownership.^50^, ^53^, ^66^, ^70^
		Perceived quality of services	• Positive perception about quality and convenience of care.^51^, ^54^, ^56^, ^57^, ^61^, ^63^, ^68^, ^77^	• Negative perception about the quality & trustworthiness of care and Inability of CBHI members to receive special treatment over non-members.^52^, ^54^, ^70^, ^77^
		Satisfaction	• Satisfaction with services.^62^, ^77^	• Dissatisfaction with provided services.^66^
	Personal predisposition	Health status	• History of illness in the household or having a chronic disease/vulnerable member of the household or poor self-reported health or recent episodes of health spending.^51^, ^59^, ^60^, ^61^, ^67^, ^69^	• No history of ill health in the household or perceived good health.^56^, ^61^, ^66^
		Affordability	• Perceived affordability of premiums/ lower premiums than OOP.^56^, ^59^, ^62^, ^69^	• Perceived unfairness in annual premiums or inability to afford premiums.^52^, ^55^, ^70^
	Socio-demographic factors (age, education level, marital status, and socio-economic status)	-	• Older age or presence of elderly people in the household.^51^, ^54^, ^57^, ^62^, ^63^, ^64^, ^76^, ^78^ • Having formal education.^56^, ^63^, ^66^, ^72^, ^74^, ^75^, ^76^, ^77^, ^78^ • Being married, divorced or widowed.^58^, ^63^, ^67^, ^69^ • Having higher socio-economic status.^56^, ^57^, ^59^, ^60^, ^61^, ^62^, ^63^, ^64^, ^67^, ^68^, ^73^, ^78^, ^79^ • Being poor.^56^, ^60^, ^67^	• Being poor.^76^, ^77^ • Higher socio-economic status.^72^, ^74^ • Older age.^74^, ^75^
Household Level	Household size & characteristics	-	• Large household size.^51^, ^58^, ^59^, ^63^, ^64^, ^66^, ^67^, ^69^, ^78^ • Female headed household.^54^ • Higher women autonomy.^78^	• large household size.^75^, ^76^ • Female headed household.^75^ • Fewer under five children.^74^
	Social capital & social cohesion	-	• Having an enrolled neighbour.^61^, ^68^ • Belonging to local community associations/solidarity groups.^50^, ^60^, ^61^, ^73^, ^77^	• Belonging to a large burial group.^68^ • Membership in a rotating saving and credit association reduced the likelihood of CBHI enrolment.^79^
Community Level	Culture & religion	-	• Being a Christian.^59^, ^60^, ^68^ • Being a Muslim, catholic or traditional woman.^75^	• Preference for herbal medicine and home healing over conventional medicine.^56^, ^70^ • Believe that joining CBHI schemes means inviting disease.^52^ • Being a protestant (woman).^75^
	Community participation	-		• Lack of community involvement/participation in the management and decision-making processes of the CBHI schemes.^50^, ^52^, ^53^
	Geographical location	-	• Being in certain regions of the country that others including urban areas.^64^, ^74^, ^75^	
Systems Level	Governance arrangement	Government/stakeholder influence	• Availability of Cash transfers and food assistance programs from donors or government.^64^, ^65^ • Donor subsidising CBHI scheme premiums.^70^	• Lack of a clear & coherent policy and legislative framework.^59^ • Inadequate technical and regulatory support from the government.^53^ • Top-down approach in CBHI policy formulation with limited input from the grassroot levels.^71^
		Management & administrative structure		• Difficulty by health facilities to access premiums causing delays in service provision.^59^ • Poor budgeting for CBHI scheme activities.^71^ • Weak leadership and management of the CBHI schemes.^53^ • Inconsistent strategies & premium levels among districts.^50^
		Content of the package and criteria of membership	• Adequate benefit package.^52^, ^61^, ^62^	• Limited benefit package.^59^, ^70^ • Exclusion of chronic disease from the benefit package.^52^ • Complicated membership criteria.^50^, ^52^, ^53^
	Financial arrangement	-	• Allowing payment by instalment.^53^	• Poor timing of premium collection.^66^, ^72^ • Lack of healthcare subsidies for the poorest to enroll.^53^, ^71^
	Service delivery arrangement	Planning and management of human resources		• Limited working hours and staff shortages.^59^ • Unresponsiveness to clients’ needs.^59^ • Misconduct by health workers (corruption).^71^
		Supplies and materials	• Availability of diagnostic laboratory services^51^, ^60^ and essential drugs.^77^	• Lack of diagnostic laboratory services and shortages of medications and essential health supplies.^50^, ^52^, ^59^, ^71^, ^72^
		Distance to health facility and patient waiting time	• Shorter waiting time.^60^ • Short distance to facility.^79^ • Long distance and travel time.^60^	• Longer distance to health facility.^70^ • High transport costs.^59^
		Choice of facility and referral systems	• Availability of referral services in the benefit package.^59^	• Lack of comprehensive services and a referral system and inability to choose health facility.^59^, ^71^

Cross-sectional studies identified key individual-level factors influencing enrolment in CBHI schemes. Awareness of CBHI was positively associated with enrolment,^57^, ^63^, ^67^, ^68^, ^76^ while limited awareness or understanding acted as a barrier.^66^, ^70^, ^74^ Positive perceptions of healthcare quality^57^, ^61^, ^63^ and satisfaction with services^62^, ^77^ facilitated enrolment, whereas dissatisfaction and poor-quality care reduced participation.^66^, ^70^

Socio-demographic characteristics associated with enrolment included older age,^57^, ^63^, ^64^, ^74^, ^75^ marital status,^63^, ^67^ higher levels of education,^63^, ^66^, ^74^, ^75^, ^76^, ^78^ and higher socio-economic status.^62^, ^63^, ^64^, ^68^, ^78^, ^79^ Poverty was a consistent barrier.^76^, ^77^ Trust in the scheme promoted uptake,^68^, ^79^ while previous experiences of fraud and perceived mismanagement reduced trust and participation.^66^, ^70^

At the household level, larger household size^63^, ^64^, ^66^, ^67^, ^78^ and female-headed households^75^ were more likely to enrol. While being a Christian is reported as an enabler at the community-level,^68^ the results became mixed in study^75^ that analysed women only. It reported that being a Muslim, Catholic or traditional woman was an enabler while being a protestant woman was a barrier. Social capital factors such as belonging to a large burial group was reported as a barrier in one study.^68^ At the systems level, barriers such as inconsistent premium levels,^50^ lack of diagnostic services,^72^ and inadequate infrastructure^79^ were reported.

Mixed-methods studies identified multiple enablers and barriers across individual, household, community, and systems levels. Personal predisposition factors such as the presence of chronic illness or recent household illness episodes were associated with higher enrolment likelihood.^59^, ^60^ Access to information through media and community sources facilitated enrolment,^51^, ^53^ although one study noted that excessive media exposure was associated with decreased likelihood of enrolment.^76^Social capital and trust, including active mobilization by enrolled members^51^ and participation in community groups such as Equb and Iddir,^60^ were associated with increased enrolment, while low community involvement and distrust in scheme management were reported as deterrents.^50^, ^53^ At the systems level, inconsistent implementation strategies, inadequate benefit packages, and weak referral systems were common challenges.^50^, ^59^ Similarly, weak leadership structures, poor budgeting practices, and lack of clear policies emerged as systemic barriers to successful scheme uptake.^59^

Qualitative studies identified contextual factors influencing CBHI enrolment. Affordability concerns were frequently reported, with premiums perceived as either too high or inequitably structured.^55^ Distrust in scheme governance emerged as a prominent barrier, driven by past instances of corruption and limited community ownership.^71^ Cultural norms were also cited as barriers, with some respondents believing that joining CBHI means inviting disease.^55^ System-level barriers such as poor health worker attitudes and practices (corruption), inadequate essential healthcare services including shortage of drugs, and weak oversight mechanisms, poor budgeting of scheme activities and system bureaucracy were also reported as critical constraints.^71^

Case-control studies reported associations between various individual and community-level factors and CBHI enrolment. Poor health status, including chronic illness, previous health expenditures, and negative self-rated health, was consistently associated with higher enrolment.^56^, ^69^, ^77^ Formal education and higher household income were also linked with increased enrolment^56^, ^72^, ^77^ except one case which reported poorer households being more likely to enrol.^56^ Positive perceptions of service quality and user satisfaction were positively associated with enrolment.^54^, ^77^ At the community level, membership in solidarity groups^73^ such Equb or Edir^77^ increased the likelihood of enrolment, whereas limited trust in community structures and scheme governance was associated with reduced participation.^56^

The case study identified involvement of community members in mobilizing more members and payment by instalment as an enablers.^53^ However, it raised key governance and implementation gaps, including inconsistencies in premium setting and widespread management inefficiencies.^53^ It also underscored the limited involvement of communities in the design and execution of schemes, which undermined local ownership and commitment. Furthermore, the study raised sustainability concerns stemming from the schemes’ heavy reliance on donor subsidies.

## *Comparative reflections and consistency across study designs*

Across the diverse methodologies, several themes consistently emerged. Awareness and access to information were identified as critical enablers in nearly all study types, with cross-sectional and mixed-methods studies emphasizing the positive role of informed communities and the negative impact of information gaps. Trust in scheme governance and effective management was a recurring theme in qualitative, mixed-methods, and cross-sectional studies, highlighting the importance of transparency and accountability. Socioeconomic status and educational attainment were strongly associated with enrolment in cross-sectional and case-control studies, though some findings noted that poorer households may also enrol. Adverse selection was a robust finding reported across case-control, cross-sectional, and mixed-methods studies, evidenced by higher enrolment among households with chronic illness, past health expenditures or older age. Cultural beliefs and religious affiliations, though less frequently explored, were noted in qualitative and cross-sectional studies as influential in shaping attitudes toward enrolment. However, divergences also emerged. While media exposure was generally associated with increased awareness, one study^76^ reported that excessive exposure may have a negative effect. Similarly, although social capital was generally linked with increased enrolment, one cross-sectional study noted that belonging to a large burial group is a barrier.^68^ Geographic and religious variations (when gender is factored in) in enrolment patterns further indicated contextual differences.

## Discussion

CBHI schemes have emerged as a key strategy to improve financial protection and access to healthcare in LMICs including SSA.^32^, ^80^ However, despite their potential, these schemes continue to face persistently low enrolment rates, which undermine their risk-pooling capacity and long-term sustainability. While a considerable body of research has explored the determinants of enrolment in CBHI schemes, much of the existing evidence originates from heterogeneous settings across Asia, South America, West Africa, and broader SSA.^28^, ^81^, ^82^, ^83^, ^84^, ^85^ There remains notable evidence gap specific to EA countries, where contextual, cultural, and systemic factors may differ substantially from other regions. As such, generating region-specific evidence is essential to inform targeted policy and programmatic interventions. This study addressed this gap by synthesizing findings from EA countries using a SEM framework to understand the multi-level drivers and barriers influencing CBHI enrolment. The findings reveal that enrolment is shaped by a complex interaction of individual, household, community, and systems-level factors. While some determinants were consistently supported across studies, others revealed contextual divergences that reflect broader challenges in implementing equitable and sustainable CBHI schemes.

### Awareness, trust, and perceptions drive enrolment decisions

At the individual level, consumer awareness and understanding of CBHI principles emerged as foundational for enrolment. Eight studies identified accurate information as an enabler,^51^, ^53^, ^56^, ^57^, ^63^, ^67^, ^68^, ^73^ while lack of awareness and poor understanding of concepts like risk pooling hindered uptake in nine studies.^51^, ^52^, ^57^, ^59^, ^66^, ^70^, ^71^, ^72^, ^74^ These findings are consistent with other studies,^28^, ^81^, ^83^, ^86^, ^87^ which found that lack of information limits understanding of the benefits of risk sharing through CBHI schemes, whereas adequate access to information enhances awareness and encourages participation. Interestingly, one study^76^ found that increased media exposure was negatively associated with enrolment, suggesting that untargeted or overwhelming messaging may create confusion rather than clarity.

Trust in the insurer and perceptions of service quality also significantly influenced enrolment. Trust, reinforced by transparent management and peer mobilization, was reported as a facilitator,^53^, ^68^, ^77^, ^79^ while distrust, often stemming from previous corruption or fraud, was a critical barrier.^50^, ^53^, ^66^, ^70^ Similarly, perceived quality of care, particularly respectful treatment and convenience, motivated enrolment,^54^, ^56^, ^57^, ^61^, ^68^ whereas perceptions of poor care or being treated similarly to non-members discouraged participation.^52^, ^54^, ^70^, ^77^ These findings are consistent with evidence from other settings.^81^, ^83^, ^88^ The explanation could be that when beneficiaries perceive that insurance membership conifers no tangible improvement in care experience, the perceived value of enrolment diminishes. This underscores the importance of aligning service delivery improvements with insurance expansion to build and sustain trust in CBHI schemes.

### Affordability and health needs shape participation in CBHI

Health status and affordability concerns were important determinants of enrolment. Households with chronic illness or recent health expenditures were more likely to enrol,^51^, ^59^, ^60^, ^61^, ^67^, ^69^ reflecting the adverse selection common in voluntary schemes. This is consistent with other studies^86^, ^89^, ^90^ that found increased enrolment among those with chronic conditions or heightened vulnerability. However, some studies also noted increased enrolment among healthier households^56^, ^61^, ^66^ which could be to mitigate future risks or that they have better understanding of CBHI and risk sharing.

In terms of affordability, high or unfair premiums were a deterrent,^52^, ^55^, ^70^ while affordability or lower cost compared to out-of-pocket payments was identified as an enabler^56^, ^59^, ^63^, ^69^ in line with other studies.^28^, ^82^ These patterns highlight the sensitivity of enrolment to pricing strategies and perceived value. When premiums are seen as disproportionate to household income or the quality of services offered, potential members may opt out. Conversely, schemes that offer clear financial protection compared to direct payments can attract higher uptake. This emphasizes the need for equitable premium structures that reflect ability to pay and perceived benefit, especially among vulnerable populations.

Socio-demographic characteristics particularly age, education, and socio-economic status (SES) emerged as important determinants of CBHI enrolment. Formal education was consistently found to be positively associated with enrolment,^56^, ^59^, ^64^, ^69^, ^73^ consistent with broader evidence.^28^, ^81^, ^82^ This could be that educated individuals are more likely to understand insurance principles, assess the benefits of membership, and navigate administrative procedures. They may also be more exposed to health promotion messaging and better positioned to influence household decisions regarding enrolment. This finding reinforces the importance of tailoring communication strategies to improve health insurance literacy, especially among populations with lower education levels.

The majority of studies found that older individuals were more likely to enrol in CBHI schemes,^51^, ^54^, ^57^, ^63^, ^64^ which aligns with the principle of adverse selection: older adults tend to have greater health needs and are thus more motivated to seek financial protection through insurance.^89^, ^90^ However, two studies^74^, ^75^ contradicted this pattern, reporting that older age was associated with lower enrolment. Possible explanations include mobility limitations, lower exposure to scheme information, or cultural preferences for traditional healing methods among older adults. These contrasting findings suggest that age-related enrolment trends may depend on the accessibility of services and how well schemes are tailored to older populations.

Most studies found that higher SES increased the likelihood of CBHI enrolment,^57^, ^62^, ^63^, ^64^, ^68^ in accordance with findings of other authors.^28^, ^82^ This reflects greater financial capacity and health system awareness. Wealthier individuals may also have more trust in formal systems and be better informed about insurance benefits as seen in other broader findings.^28^, ^81^, ^82^ However, two studies^72^, ^74^ found higher SES to be negatively associated with enrolment. This could be that wealthier individuals may prefer to pay out of pocket or seek private insurance options that offer more perceived value, flexibility, or status. These divergent findings suggest that high SES does not uniformly predict enrolment and that choice behaviour may differ across socio-economic strata. Furthermore, varying definitions and measurements of SES across studies may contribute to these differences.

Evidence on the relationship between poverty and enrolment was mixed. Some studies^76^, ^77^ found poverty to be a barrier, suggesting affordability issues and competing household priorities that limited the feasibility of premium payments. In contrast, other studies^56^, ^60^, ^67^ reported that poor households did enrol in CBHI schemes, possibly due to targeted subsidies, strong community outreach, or a perceived high value for money. These contrasting findings highlight that the effect of poverty on enrolment is context-dependent and shaped by scheme design features such as premium levels, subsidy arrangements, and the strength of community engagement mechanisms and how poverty is measured.

### Sociocultural and community dynamics influence CBHI uptake

Household characteristics such as larger household size,^51^, ^58^, ^59^, ^63^, ^64^, ^66^, ^67^, ^69^ female-headed households^51^, ^78^ and higher women autonomy^74^ were generally associated with higher enrolment, perhaps due to increased care needs and greater health-seeking behaviour. This is consistent with broader findings in SSA and LMICs.^81^, ^91^ However, other studies found large households^52^, ^74^, ^75^ and female-headed households^75^ were less likely to enrol, suggesting that financial strain and gendered vulnerabilities may counteract potential benefits.

Further on household characteristics, one study which reported on the number of under-five children found that having fewer under-five children was linked to lower enrolment,^78^ possibly due to reduced perceived health risks and service needs, leading to less motivation to join CBHI schemes.

Social capital emerged as a critical enabler. Belonging to local associations or having an enrolled neighbour increased enrolment,^61^, ^73^, ^77^ reinforcing the value of peer influence and social learning. These findings are in line with evidence from other studies^81^, ^82^, ^88^, ^92^ which highlight how community networks and interpersonal trust can facilitate scheme uptake. Conversely, belonging to some traditional solidarity groups, such as rotating savings groups^79^ or burial societies,^52^ was associated with lower enrolment, potentially reflecting alternative risk-sharing arrangements or scepticism toward formal insurance.

Cultural and religious beliefs also shaped attitudes toward CBHI. Being a Christian was generally associated with increased enrolment as cited in three studies.^59^, ^60^, ^68^ This could be because Christian communities often have stronger social networks, greater trust in organized schemes, and higher exposure to health education through church-based initiatives. However, the results were mixed when gender factors were considered. For example, one study^75^ found that being a Muslim, Catholic or traditional woman increased enrolment, while being Protestant was a barrier. These patterns suggest that religious norms and gender roles may interact to shape health seeking behaviour among women. Regarding culture, certain cultural practices such as reliance on herbal medicine^56^, ^70^ or beliefs that CBHI invites illness^52^ posed barriers to CBHI enrolment. Such practices reflect deep-rooted health beliefs that may undermine the perceived need for formal health insurance. In communities where traditional medicine is trusted and widely used, CBHI may be viewed as irrelevant or unnecessary. Similarly, fatalistic beliefs such as the notion that preparing for illness through insurance attracts misfortune, can discourage uptake. These cultural perceptions highlight the importance of culturally sensitive health education and engagement strategies that respect local beliefs while promoting the benefits of CBHI.

### System-Level factors can either enable or undermine CBHI effectiveness

System-level structures, including governance, financing, benefit design, and service delivery arrangements, were found to be decisive in shaping CBHI uptake. Weak governance structures, poor regulatory oversight, and top-down decision-making approaches were frequently cited as barriers.^53^, ^59^, ^71^ These limitations can erode community trust, reduce accountability, and lead to misaligned scheme designs that fail to meet local needs. In contrast, external support such as donor subsidies or social protection programs^64^, ^65^, ^70^ helped reduce financial barriers and incentivize enrolment. These findings are consistent with evidence from other studies,^28^, ^81^, ^82^, ^88^ highlighting the need for strong institutional frameworks and locally responsive policies to ensure both uptake and durability of CBHI schemes.

Design and operational issues were also reported to influence enrolment. Administrative inefficiencies such as delayed premium reimbursement,^59^ poor budgeting and inconsistent strategies,^71^ undermined confidence in scheme reliability. Schemes that excluded chronic diseases,^52^ offered limited benefits,^59^, ^70^ or imposed complex membership criteria^53^ faced low uptake. These features reduce the perceived value of enrolment and create access barriers, especially for vulnerable groups. However, flexible payment options^53^ and comprehensive benefit packages^61^, ^62^ were seen as strong enablers which align with other findings in SSA and broader LMICs.^28^, ^81^, ^93^ These elements enhance financial protection and perceived fairness, making schemes more attractive and inclusive.

Service delivery challenges were recurrent. Frequent drug stockouts, staff shortages, and health worker misconduct^59^, ^71^ undermined trust in the health system and reduced CBHI uptake. Similarly, long distances to facilities, high transportation costs, and extended waiting times^59^, ^70^ were common deterrents while proximity to health facilities was cited as an enabler. Interestingly, one study^60^ reported that long distances increased enrolment, contradicting prevailing literature. This may reflect the perceived value of services at distant facilities or the presence of reliable transport infrastructure that offsets distance barriers. Further research is needed to unpack the contextual drivers of this finding.

### Lessons from beyond East Africa: Rwanda and other LMICs

Rwanda is often cited as a success story, where political will, mandatory enrolment, and strong governance structures have enabled high coverage and sustainability.^36^ Unlike many voluntary CBHI schemes, Rwanda’s approach integrates CBHI into national health financing policy, with robust subsidies and standardized benefit packages.^36^ These institutional features contrast sharply with the fragmented and under-resourced schemes found in many EA countries.

While this review focused on EA, broader evidence from other LMICs reinforces and extends these findings. Evidence from West Africa and SSA at large underscores the importance of legislative backing, premium subsidies for the poor, and digitalized systems to streamline enrolment and claims processing.^94^, ^95^, ^96^ These cases suggest that successful scale-up of CBHI requires a strong policy foundation, adequate resourcing, and sustained government commitment, lessons that can inform the design and strengthening of CBHI in EA.

### Strengths and limitations

This study has several strengths, including its comprehensive approach to examining CBHI enrolment determinants using a SEM framework. The scoping review methodology and transparent reporting processes used contribute to the reliability and validity of the findings. Additionally, although only peer-reviewed articles were included in the final analysis, grey literature was consulted to inform the study’s conceptual background, framing, and scope, thereby offering contextual insights and enhancing its comprehensiveness.

However, the findings should be interpreted in light of several limitations. First, there is potential for thematic overlap in the grouping of findings across socio-ecological levels, which may have influenced the clarity of categorization. Second, the exclusion of non-English language studies may have led to the omission of relevant literature, potentially introducing language bias. Third, the geographical distribution of included studies was uneven, with a majority conducted in Ethiopia (n = 19), and fewer from Tanzania (n = 7), Uganda (n = 3), and Kenya (n = 1), while no studies were identified from the remaining 8 eligible EA countries. This imbalance limits the generalizability of the findings across the subregion. In addition, the dominance of cross-sectional designs (n = 16), compared to case-control (n = 6), mixed-methods (n = 5), and qualitative studies (n = 2), may have constrained both the contextual depth and the capacity to infer causality. Factors such as cultural beliefs and trust were more commonly explored in qualitative and mixed-methods studies, suggesting that reliance on quantitative designs may underrepresent key enrolment determinants. These limitations underscore the need for more diverse, inclusive, and methodologically varied research to strengthen the certainty and applicability of CBHI evidence across EA.

## Conclusions

This study offers a comprehensive synthesis of the determinants of enrolment in CBHI schemes in EA, using a SEM framework. The evidence highlights that enrolment decisions are shaped by a complex interplay of factors across individual, household, community, and system levels. Key enablers include good consumer awareness and insurance literacy, trust in scheme governance, perceived quality and respectful care, affordable and flexible premiums, formal education, and social capital through community networks and peer influence. System-level support such as strong governance, donor subsidies, comprehensive benefit packages, accessible health services, and efficient administration also promote enrolment. Conversely, persistent barriers include low awareness, distrust due to past mismanagement, perceptions of poor care, unaffordable premiums, and exclusionary or complex scheme designs. Poverty, competing household priorities, cultural beliefs favoring traditional medicine, weak governance, administrative inefficiencies and service delivery challenges further hinder enrolment and threaten scheme viability. To achieve scale and sustainability, policy makers must adopt a holistic approach that reinforces governance structures, standardizes benefit packages, improves service quality, creating awareness and meaningfully includes communities in scheme design and oversight. Integrating these priorities can enhance equity, increase uptake, and bring EA’s countries closer to UHC.

## List of abbreviations

**Table:** 

AfCDC	Africa Centres for Disease Control and Prevention
AfDB	African Development Bank
AIM	Africa Index Medicus
CBHI	Community-based Health Insurance
CHF	Community Health Fund
CHE	Catastrophic Health Expenditure
CSOs	Civil Society Organizations
EA	East Africa
EAHRC	East African Health Research Council
ETC	Et Cetera
FCDO	Foreign Commonwealth and Development Office
FDGs	Focused Group Discussions
FFS	Fee For Service
Govt	Government
HHs	Households
HPPF	Health Policy Planning and Financing
HTA	Health Technology Assessment
IDLs	Individuals
KIIS	Key Informant Interviewees
LMICs	Low-and Middle-Income Countries
LSE	The London School of Economics and Political Science
LSHTM	London School of Hygiene and Tropical Medicine
OOP	Out-of-Pocket Payments
PPMs	Provider Payment Mechanisms
PRISMA-ScR	Preferred Reporting Items for Systematic Reviews and Meta-Analysis Extension for Scoping Reviews
SEM	Socioecological Model
SHI	Social Health Insurance
SSA	Sub-Saharan Africa
UHC	Universal Health Coverage
WB	World Bank
WHO	World Health Organization
WTE	Willingness-to-Enroll
WTP	Willingness to Pay

## Ethics approval and consent to participate

Ethics approval was waived by the Ethics Committee of the London School of Hygiene and Tropical Medicine (request number: 29328) on the basis that this is a review of the literature.

## Funding

Not applicable

## Consent for publication

Not applicable

## Authors' contributions

RL conceptualized the study, conducted the research, analyzed the data, and drafted the first version of the manuscript. SS supervised the study, provided comprehensive feedback, and suggested areas for improvement. OOO provided scientific guidance for writing the manuscript, critically reviewed all the drafts of the manuscript, and provided valuable insights to restructure all drafts. All authors read and provided significant inputs into all drafts of the manuscript, agreed to be accountable for all aspects of the work and approved the final draft of the manuscript for publication.

## Declaration of Competing Interest

The authors declare that they have no known competing financial interests or personal relationships that could have appeared to influence the work reported in this paper.

## Data Availability

The data supporting the findings of this study, including all generated tables and the search string used in the study are available as supplementary material.

## Appendix A Supplementary material

Supplementary material 1The actual search strategy used

Supplementary material 2Summary of results for each study

## Footnotes

Appendix A Supplementary data associated with this article can be found in the online version at doi:10.1016/j.ghrp.2026.01.002.
