# Supplementary material for: Informing policy through evidence: A scoping review of factors that influence enrolment in community-based health insurance in East Africa
Source: Glob Health Res Policy. 2026 Feb 3;11(1):8–19. doi: 10.1016/j.ghrp.2026.01.002 (PMC13017189; doi:10.1016/j.ghrp.2026.01.002)
Supplement: Supplementary file 1 — Supplementary material 1The actual search strategy used [file mmc1.docx]

**DETAILED ELECTRONIC SEARCH STRATEGY**

| **Ovid MEDLINE** | | | |
| --- | --- | --- | --- |
| S. No | KEY CONCEPT | MEDLINE SEARCH **1** TERMS | RESULTS |
| #1 | Enrolment | (enrol* OR enroll* OR registration OR enlistment OR admission OR participation OR membership OR joining OR sign-up OR enrollee* OR subscriber* OR insured OR beneficiary* OR recipient* OR uptake OR "access fees" OR pay).mp. | 2,135,472 |
| #2 | Community-based health insurance | (CBHI OR "community based health insurance" OR "community based health financing" OR "community health risk sharing" OR "community health schemes" OR "cooperative health insurance" OR "customary health financing" OR "grassroots health scheme*" OR "indigenous health insurance" OR "informal health insurance" OR "informal health risk-sharing arrangement*" OR "local health insurance program*" OR "micro health insurance" OR "mutual aid health coverage" OR "mutual health organi?ation" OR "non-commercial health insurance" OR "non-profit health insurance" OR "pro-poor health insurance" OR "rural health financ*" OR "rural health insurance" OR "self-help health insurance" OR "social capital health insurance" OR "social health protection" OR "solidarity-based health insurance" OR "solidarity-based health protection" OR "unofficial health coverage" OR "village health insurance" OR "voluntary health insurance").mp. | 6,482 |
| #3 | East Africa | ("east* Africa*" OR "horn of Africa" OR "East* Africa* region" OR "East* Africa* countr*" OR "East* Africa* nation*" OR "East* Africa* state*" OR "East* Africa* societ*" OR "East* Africa* culture*" OR Kenya OR Tanzania OR Uganda OR Rwanda OR Burundi* OR Ethiopia* OR Eritrea* OR Somali* OR Djibouti OR Comoros OR Sudan* OR "South* Sudan*").mp. | 261,783 |
| #4 | LIMITS | 1 AND 2 AND 3 | 122 |
| #5 |  | Limit 4 to (humans and yr="2000 - 2023") | 113 |
|  | | | |
| S. No | KEY CONCEPT | MEDLINE SEARCH **2** TERMS | RESULTS |
| #1 | Enrolment | (enrol* OR enroll* OR registration OR enlistment OR admission OR participation OR membership OR joining OR sign-up OR enrollee* OR subscriber* OR insured OR beneficiary* OR recipient* OR uptake OR "access fees" OR pay).mp. | 205,118 |
| #2 | Community-based health insurance | (CBHI OR "community based health insurance" OR "community based health financing" OR "community health risk sharing" OR "community health schemes" OR "cooperative health insurance" OR "customary health financing" OR "grassroots health scheme*" OR "indigenous health insurance" OR "informal health insurance" OR "informal health risk-sharing arrangement*" OR "local health insurance program*" OR "micro health insurance" OR "mutual aid health coverage" OR "mutual health organi?ation" OR "non-commercial health insurance" OR "non-profit health insurance" OR "pro-poor health insurance" OR "rural health financ*" OR "rural health insurance" OR "self-help health insurance" OR "social capital health insurance" OR "social health protection" OR "solidarity-based health insurance" OR "solidarity-based health protection" OR "unofficial health coverage" OR "village health insurance" OR "voluntary health insurance").mp. | 548 |
| #3 | East Africa | ("east* Africa*" OR "horn of Africa" OR "East* Africa* region" OR "East* Africa* countr*" OR "East* Africa* nation*" OR "East* Africa* state*" OR "East* Africa* societ*" OR "East* Africa* culture*" OR Kenya OR Tanzania OR Uganda OR Rwanda OR Burundi* OR Ethiopia* OR Eritrea* OR Somali* OR Djibouti OR Comoros OR Sudan* OR "South* Sudan*").mp. | 23,377 |
| #4 | LIMITS | 1 AND 2 AND 3 | 33 |
| #5 |  | Limit 4 to (humans and english language and yr="2023 - 2025") | 29 |
| **Ovid EMBASE** | | | |
| S. No | KEY CONCEPT | EMBASE SEARCH **1** TERMS | RESULTS |
| #1 | Enrolment | (enrol* OR enroll* OR registration OR enlistment OR admission OR participation OR membership OR joining OR sign-up OR enrollee* OR subscriber* OR insured OR beneficiary* OR recipient* OR uptake OR "access fees" OR pay).mp. | 3,268,914 |
| #2 | Community-based health insurance | (CBHI OR "community based health insurance" OR "community based health financing" OR "community health risk sharing" OR "community health schemes" OR "cooperative health insurance" OR "customary health financing" OR "grassroots health scheme*" OR "indigenous health insurance" OR "informal health insurance" OR "informal health risk-sharing arrangement*" OR "local health insurance program*" OR "micro health insurance" OR "mutual aid health coverage" OR "mutual health organi?ation" OR "non-commercial health insurance" OR "non-profit health insurance" OR "pro-poor health insurance" OR "rural health financ*" OR "rural health insurance" OR "self-help health insurance" OR "social capital health insurance" OR "social health protection" OR "solidarity-based health insurance" OR "solidarity-based health protection" OR "unofficial health coverage" OR "village health insurance" OR "voluntary health insurance").mp. | 8,503 |
| #3 | East Africa | ("east* Africa*" OR "horn of Africa" OR "East* Africa* region" OR "East* Africa* countr*" OR "East* Africa* nation*" OR "East* Africa* state*" OR "East* Africa* societ*" OR "East* Africa* culture*" OR Kenya OR Tanzania OR Uganda OR Rwanda OR Burundi* OR Ethiopia* OR Eritrea* OR Somali* OR Djibouti OR Comoros OR Sudan* OR "South* Sudan*").mp. | 381,974 |
| #4 | LIMITS | 1 AND 2 AND 3 | 139 |
| #5 |  | Limit 4 to (humans and yr="2000 - 2023") | 126 |
|  | | | |
| S. No | KEY CONCEPT | EMBASE SEARCH **2** TERMS | RESULTS |
| #1 | Enrolment | (enrol* OR enroll* OR registration OR enlistment OR admission OR participation OR membership OR joining OR sign-up OR enrollee* OR subscriber* OR insured OR beneficiary* OR recipient* OR uptake OR "access fees" OR pay).mp. | 158,740 |
| #2 | Community-based health insurance | (CBHI OR "community based health insurance" OR "community based health financing" OR "community health risk sharing" OR "community health schemes" OR "cooperative health insurance" OR "customary health financing" OR "grassroots health scheme*" OR "indigenous health insurance" OR "informal health insurance" OR "informal health risk-sharing arrangement*" OR "local health insurance program*" OR "micro health insurance" OR "mutual aid health coverage" OR "mutual health organi?ation" OR "non-commercial health insurance" OR "non-profit health insurance" OR "pro-poor health insurance" OR "rural health financ*" OR "rural health insurance" OR "self-help health insurance" OR "social capital health insurance" OR "social health protection" OR "solidarity-based health insurance" OR "solidarity-based health protection" OR "unofficial health coverage" OR "village health insurance" OR "voluntary health insurance").mp. | 764 |
| #3 | East Africa | ("east* Africa*" OR "horn of Africa" OR "East* Africa* region" OR "East* Africa* countr*" OR "East* Africa* nation*" OR "East* Africa* state*" OR "East* Africa* societ*" OR "East* Africa* culture*" OR Kenya OR Tanzania OR Uganda OR Rwanda OR Burundi* OR Ethiopia* OR Eritrea* OR Somali* OR Djibouti OR Comoros OR Sudan* OR "South* Sudan*").mp. | 35,103 |
| #4 | LIMITS | 1 AND 2 AND 3 | 41 |
| #5 |  | Limit 4 to (humans and english language and yr="2023 - 2025") | 36 |
| **SCOPUS** | | | |
| S. No | KEY CONCEPT | SCOPUS SEARCH **1** TERMS | RESULTS |
| #1 | Enrolment  Community-based health insurance  East Africa | (TITLE-ABS-KEY(enrol* OR enroll* OR registration OR enlistment OR admission OR participation OR membership OR joining OR "sign-up" OR enrollee* OR subscriber* OR insured OR beneficiary* OR recipient* OR uptake OR "access fees" OR pay))  AND  (TITLE-ABS-KEY(CBHI OR "community based health insurance" OR "community based health financing" OR "community health risk sharing" OR "community health schemes" OR "cooperative health insurance" OR "customary health financing" OR "grassroots health scheme*" OR "indigenous health insurance" OR "informal health insurance" OR "informal health risk-sharing arrangement*" OR "local health insurance program*" OR "micro health insurance" OR "mutual aid health coverage" OR "mutual health organi?ation" OR "non-commercial health insurance" OR "non-profit health insurance" OR "pro-poor health insurance" OR "rural health financ*" OR "rural health insurance" OR "self-help health insurance" OR "social capital health insurance" OR "social health protection" OR "solidarity-based health insurance" OR "solidarity-based health protection" OR "unofficial health coverage" OR "village health insurance" OR "voluntary health insurance"))  AND  (TITLE-ABS-KEY("east* Africa*" OR "horn of Africa" OR "East* Africa* region" OR "East* Africa* countr*" OR "East* Africa* nation*" OR "East* Africa* state*" OR "East* Africa* societ*" OR "East* Africa* culture*" OR Kenya OR Tanzania OR Uganda OR Rwanda OR Burundi* OR Ethiopia* OR Eritrea* OR Somali* OR Djibouti OR Comoros OR Sudan* OR "South* Sudan*"))  AND  (PUBYEAR > 1999 AND PUBYEAR < 2024) | 129 |
|  | | | |
| S. No | KEY CONCEPT | SCOPUS SEARCH **2** TREMS | RESULTS |
| #1 | Enrolment  Community-based health insurance  East Africa | (TITLE-ABS-KEY(enrol* OR enroll* OR registration OR enlistment OR admission OR participation OR membership OR joining OR "sign-up" OR enrollee* OR subscriber* OR insured OR beneficiary* OR recipient* OR uptake OR "access fees" OR pay))  AND  (TITLE-ABS-KEY(CBHI OR "community based health insurance" OR "community based health financing" OR "community health risk sharing" OR "community health schemes" OR "cooperative health insurance" OR "customary health financing" OR "grassroots health scheme*" OR "indigenous health insurance" OR "informal health insurance" OR "informal health risk-sharing arrangement*" OR "local health insurance program*" OR "micro health insurance" OR "mutual aid health coverage" OR "mutual health organi?ation" OR "non-commercial health insurance" OR "non-profit health insurance" OR "pro-poor health insurance" OR "rural health financ*" OR "rural health insurance" OR "self-help health insurance" OR "social capital health insurance" OR "social health protection" OR "solidarity-based health insurance" OR "solidarity-based health protection" OR "unofficial health coverage" OR "village health insurance" OR "voluntary health insurance"))  AND  (TITLE-ABS-KEY("east* Africa*" OR "horn of Africa" OR "East* Africa* region" OR "East* Africa* countr*" OR "East* Africa* nation*" OR "East* Africa* state*" OR "East* Africa* societ*" OR "East* Africa* culture*" OR Kenya OR Tanzania OR Uganda OR Rwanda OR Burundi* OR Ethiopia* OR Eritrea* OR Somali* OR Djibouti OR Comoros OR Sudan* OR "South* Sudan*"))  AND  (LIMIT-TO(LANGUAGE, "English"))  AND  (PUBDATETXT > 2023-06-26 AND PUBDATETXT < 2025-06-15) | 58 |
| **AFRICAN INDEX MEDICUS (AIM)** | | | |
| S. No | KEY CONCEPT | AIM SEARCH **1** TERMS | RESULTS |
| #1 | Enrolment  Community-based health insurance  East Africa | ("enrol*" OR "enroll*" OR registration OR enlistment OR admission OR participation OR membership OR joining OR "sign-up" OR enrollee* OR subscriber* OR insured OR beneficiary* OR recipient* OR uptake OR "access fees" OR pay)  AND  ("CBHI" OR "community based health insurance" OR "community based health financing" OR "community health risk sharing" OR "community health schemes" OR "cooperative health insurance" OR "customary health financing" OR "grassroots health scheme*" OR "indigenous health insurance" OR "informal health insurance" OR "informal health risk-sharing arrangement*" OR "local health insurance program*" OR "micro health insurance" OR "mutual aid health coverage" OR "mutual health organi?ation" OR "non-commercial health insurance" OR "non-profit health insurance" OR "pro-poor health insurance" OR "rural health financ*" OR "rural health insurance" OR "self-help health insurance" OR "social capital health insurance" OR "social health protection" OR "solidarity-based health insurance" OR "solidarity-based health protection" OR "unofficial health coverage" OR "village health insurance" OR "voluntary health insurance")  AND  ("east* Africa*" OR "horn of Africa" OR "East* Africa* region" OR "East* Africa* countr*" OR "East* Africa* nation*" OR "East* Africa* state*" OR "East* Africa* societ*" OR "East* Africa* culture*" OR Kenya OR Tanzania OR Uganda OR Rwanda OR Burundi* OR Ethiopia* OR Eritrea* OR Somali* OR Djibouti OR Comoros OR Sudan* OR "South* Sudan*") | 005 |
| S. No | KEY CONCEPT | AIM SEARCH **2** TERMS | RESULTS |
| #1 | Enrolment  Community-based health insurance  East Africa | ("enrol*" OR "enroll*" OR registration OR enlistment OR admission OR participation OR membership OR joining OR "sign-up" OR enrollee* OR subscriber* OR insured OR beneficiary* OR recipient* OR uptake OR "access fees" OR pay)  AND  ("CBHI" OR "community based health insurance" OR "community based health financing" OR "community health risk sharing" OR "community health schemes" OR "cooperative health insurance" OR "customary health financing" OR "grassroots health scheme*" OR "indigenous health insurance" OR "informal health insurance" OR "informal health risk-sharing arrangement*" OR "local health insurance program*" OR "micro health insurance" OR "mutual aid health coverage" OR "mutual health organi?ation" OR "non-commercial health insurance" OR "non-profit health insurance" OR "pro-poor health insurance" OR "rural health financ*" OR "rural health insurance" OR "self-help health insurance" OR "social capital health insurance" OR "social health protection" OR "solidarity-based health insurance" OR "solidarity-based health protection" OR "unofficial health coverage" OR "village health insurance" OR "voluntary health insurance")  AND  ("east* Africa*" OR "horn of Africa" OR "East* Africa* region" OR "East* Africa* countr*" OR "East* Africa* nation*" OR "East* Africa* state*" OR "East* Africa* societ*" OR "East* Africa* culture*" OR Kenya OR Tanzania OR Uganda OR Rwanda OR Burundi* OR Ethiopia* OR Eritrea* OR Somali* OR Djibouti OR Comoros OR Sudan* OR "South* Sudan*") | 001 |
| **GREY LITERATURE** | | | |
| S. No | SOURCE | KEY CONCEPT | RESULTS |
| #1 | Google Search 1  June 27 2023 | Enrolment  Community-based health insurance  East Africa | 128 |
| #2 | Google Search 2  June 14 2025 | Enrolment  Community-based health insurance  East Africa | 12 |
| #3 | Google Scholar Search 1  June 27 2023 | Enrolment  Community-based health insurance  East Africa | 80 |
| #4 | Google Scholar Search 2  June 14 2025 | Enrolment  Community-based health insurance  East Africa | 7 |
| **SELECTED INSTITUTIONAL WEBSITES** | | | |
| S. No | INSTITUTION | URL | RESULTS |
| #1 | WHO | <https://who.int> | 2 |
| #2 | AFRICA CDC | <https://africacdc.org> | 3 |
| #3 | East Africa Health Research Commission (EAHRC) | [https://eahrc.org](https://eahrc.org/) | 3 |
