# Supplementary material for: Informing policy through evidence: A scoping review of factors that influence enrolment in community-based health insurance in East Africa
Source: Glob Health Res Policy. 2026 Feb 3;11(1):8–19. doi: 10.1016/j.ghrp.2026.01.002 (PMC13017189; doi:10.1016/j.ghrp.2026.01.002)
Supplement: Supplementary file 2 — Supplementary material 2Summary of results for each study [file mmc2.docx]

# Appendix 2: Summary of results

| **No** | **Author** | **Country** | **Outcome Measured** | **Determinants of enrollment to CBHI Schemes** | |
| --- | --- | --- | --- | --- | --- |
|  |  |  |  | **Enablers of Enrollment to CBHI** | **Barriers of enrollment to CBHI** |
| 1 | Abdilwohab *et al.*, 2021 | Southern Ethiopia | Enrollment | - Majority of enrolled participants who found CBHI premiums affordable are affiliated with social solidarity groups such as idir (funeral savings group) and equb (traditional savings). | - Beneficiaries have Trust issues in the CBHI management team due to history of fraud. - Low enrollment attributed to poor community involvement and limited integration with existing government structures. - Weak leadership and lack of commitment of district-level managers. - Inconsistent CHBI implementation strategies and variation in premium levels across different districts. - Fraud and abuse of premium by district coordinators and health officials. - The requirement of at least 50% membership before initiation of services deterred enrollment |
| 2 | Atafu and Kwon, 2018 | Northwest Ethiopia | Enrollment | - Households that are Informed about CBHI have higher probability of enrollment than non-informed. - Household heads with good perception about the quality of public health services have high odds of enrollment to CBHI. - Individuals with poor self-rated health or have chronic disease have high odds of enrollment than their health counterparts, suggesting adverse selection. - Older households have higher odds of enrollment. - Larger household size (over 5) has higher odds of enrollment than smaller ones (under 5). - Availability of laboratory services like blood glucose tests increase enrollment. | - Lack of awareness about CBHI scheme prevented non-members from enrolling, with insufficient awareness campaigns cited by individuals as cause. - The concept of health insurance and risk pooling is low. |
| 3 | Basaza, Criel and Van der Stuyft, 2008 | Uganda | Enrollment | - Availability of treatment for all diseases encouraged enrollment. | - Contents of sensitization not tailored to/align with the core principles of CBHI. - Poor understanding of the concept of risk pooling by community, who see no benefit if they do not fall sick. - Quality concerns raised by members and non-members about dirty hospital environment and long queue. - Inability to pay for membership especially large families. - Cultural belief that joining CBHI means inviting disease. - Scheme members not involved in decision making which discouraged enrollment. - Exclusion of treatment of chronic diseases from the benefit package discouraged enrollment. - Difficulty to raise the adequate number or group (60%) before enrollment. - Lack of modern equipment and prescribed medicines discouraged enrollment. |
| 4 | Basaza, Criel and Van der Stuyft, 2007 | Uganda | Enrollment | - Understanding of the concept of insurance made some members invite new members. - Scheme members involved in mobilization of more scheme members. - Payment by instalments was an enabling factor for enrollment. | - Lack of information and poor understanding of the notion of CBHI. - Lack of trust in the local financial institutions after previous negative experience. - Low level of community involvement in management of hospital based CBHI schemes. - Lack of clear and coherent policy and legislative framework coupled with lack of technical and regulatory support. - Lack of practical expertise by MOH and donors in setting up CBHI. - Difficulty for communities to raise 60% membership or 100 families per village. - Inability to pay and lack of subsidies for the poor discouraged enrollment. |
| 5 | Chanie and Ewunetie, 2020 | North Gondar, Ethiopia | Membership | - Majority of households with positive perception about CBHI were enrolled to CBHI scheme. - Enrollment increases with age. - Female household head increases chances of enrollment | - Households’ heads with poor perception about CBHI schemes were less likely to enroll compared to those who do not think so. |
| 6 | Demissie and Atnafu, 2021 | Northwest Ethiopia | Enrollment |  | - Participants and non-participants complained about high premiums which keep increasing annually. - Participants and non-participants complained about quality of the services including long waiting times. |
| 7 | Fite *et al.*, 2021 | Western Ethiopia | Enrollment | - Participants with good awareness of CBHI were more likely to be enrolled to CBHI scheme compared to those with poor awareness. - Participants who perceived quality of care to be high were more likely to be enrolled to CBHI scheme compared to those that do not perceive so. - Participants who perceived premiums to be cheap were more likely to enroll to CBHI scheme than those who perceived premiums to be expensive. - Participants who perceived their health to be good were more likely to be enrolled to CBHI scheme than those who perceive their health to be poor, contrary to popular literature and beliefs. - Households from poor and medium wealth index were more likely to be enrolled to CBHI scheme than the rich wealth index. - Participants who were educated (college or above) were more likely to be enrolled to CBHI scheme than those who are not educated. | - Households who prefer traditional medicine/home-healing to modern medicine were less likely to be enrolled to CBHI scheme than those whose treatment choice is modern medicine. |
| 8 | Kagaigai *et al.*, 2021 | Tanzania | Enrollment | - Increase in knowledge increased the chances households’ enrollment to iCHF. - Enrollment to iCHF increased with increase in quality and convenience. - Odds of enrollment to iCHF increased with increase in income. | - Odds of enrollment to iCBF is lower for younger people (18-49) compared to older people (60 years and above). |
| 9 | Kapologwe *et al.*, 2017 | Tanzania | Enrollment | - Married, separated, widow or divorced households were more likely to be enrolled and re-enrolled to a CBHI scheme than single ones. - Households with six or more members were more likely to be enrolled to CBHI scheme than those with 5 or less members. |  |
| 10 | Macha *et al.*, 2014 | Tanzania | Membership | - The common view by that premiums were less than the sum of out-of-pocket payment incentivised enrollment. - Having vulnerable family member was a push factor into CBHI enrollment, suggesting adverse selection. - Wealthy members were more likely to be enrolled to CBHI scheme than the poorest in the community. - People doing business, farming or own cattle were better seen to be able to pay premiums compared to those without. - Larger families were more likely to be CBHI members, with each added member further increasing the chances of enrollment. - Christians were more likely to be members of a CBHI scheme than other religious groups. - Inclusion of referral services in Mbulu encouraged enrollment. | - Poor understanding of CBHI concept where members thought their premiums will remain available for them if they did not seek care. - The government exemption policy which offered free PHC services discouraged enrollment to CBHI. - Barriers in accessing government matching grants for purchasing drugs and improving quality impacted efforts to improve CBHI services. - Limited benefit package with services offered in one facility discouraged well-off people from joining the scheme, as they can afford paying OOP. - Limited working hours at the facility affected membership. - Low quality of health care services at public health facilities (shortage of drugs, diagnostic equipment, and long waiting times) discouraged people from enrolling. - Transport costs to facility discouraged enrollment. - Expensive referral services which are not covered by CBHI scheme. |
| 11 | Mebratie *et al.*, 2015 | Ethiopia | Enrollment | - Recent episodes of out-of-pocket spending on outpatient services motivated individuals to enroll. - The productive safety net program (PSNP) encouraged the enrollment of the poorest (chronically food insecure) participants. - Present or previous community leadership role enhanced enrollment. - Belonging to social network groups such as Iddir (funeral association) or Iqqub (informal savings) raised awareness about CBHI and subsequently increased enrollment. - Orthodox Christians were more likely to enroll to CBHI scheme than other religions. - Availability of blood testing equipment in the nearest facility increased the chances of enrollment by 30%. - Contrary to popular beliefs, long distance and increased travel time to health facility increased enrollment to CBHI schemes. | - Long waiting times decreased enrollment in the CBHI scheme. |
| 12 | Mirach, Demissie and Biks, 2019 | Northwest Ethiopia | Implementation | - Participants with good perception of quality of services have high chances of enrolment compared to the ones with contrary perception. - Households with chronic disease more likely to enroll than healthy ones, an adverse selection. - Wealthy households were more likely to enroll than the poor ones. - Household participation in local informal associations (credit and savings) is positively associated with CBHI enrollment. - Households with the perception that benefit package is adequate were more likely to enroll than those who perceive it as inadequate. | - Households with perceived good health are less likely to enroll than those with poor perceived health. |
| 13 | Modest, Ngowi and Katalambula, 2021 | Dodoma Tanzania | Enrollment | - Households more satisfied with services were more likely to enroll than those who were dissatisfied. - Households with more than one elders (aged 60 and above) were more likely to enroll in the iCHF than families with no elder, suggesting adverse selection. - Wealthiest households more likely to enroll than the poor ones. - Households who received motivational benefits at the health facility were more likely to enroll than those who did not. |  |
| 14 | Mollel, Mgumia and Nkonoki, 2016 | Central Tanzania | Membership | - Awareness about CHF positively influenced membership. - Quality of services provided by the scheme positively associated with membership though statistically insignificant. - Premium affordability positively associated with CHF membership though statistically insignificant. - Increasing age positively influence enrollment though not statistically significant. - Monthly income positively associated with CHF membership. - Education positively influence CHF membership. - Married people more likely to enroll compared to the single ones, suggesting marriage associated with children and thus responsibility. - Large household (over 4) associated with enrollment compared with low household size. |  |
| 15 | Moyehodie, Mulugeta and Yilema, 2022 | Ethiopia | Enrollment | - Older households (35-74 years) more likely to enroll to CBHI scheme than younger ones (15-34 years), suggesting adverse selection. - Richer households (including those who own land for agriculture, mobile telephones, livestock herds or farm animals) more likely to enroll to CBHI scheme than the poor ones. - Large households (4-6 members) more likely to enroll than smaller ones (1-3 members). - Households with more children more likely to enroll than those with no children. - Households who received cash of food from the safety net programme more likely to enroll than those who did not receive. | - Households from the Afar region, Oromia region, Somali region, Benishangul administration, Dire Dawa city administration were less likely to enroll than those in Tigray region. |
| 16 | Mussa, Agegnehu and Nshakira-Rukundo, 2022 | Amhara Region Ethiopia | Enrollment | - Participation in the PNSP’s conditional cash transfers programme increased enrollment to CBHI among participants. |  |
| 17 | Nageso, Tefera and Gutema, 2020 | Ethiopia | Enrollment | - Educated participants (secondary level and above) were more likely to be enrolled in CBHI scheme than uneducated ones. - Large family size (5 or more) was more likely to be enrolled to CBHI than their smaller counterparts (5 or less). | - Respondents not informed about CBHI less likely to enroll than those who were informed. - Household heads who did not trust scheme management were less likely to be enrolled than those who trusted management. - Enrollment for participants who were dissatisfied with services in the near facility was lower than that for those who were satisfied with scheme. - Less enrollment status among participants who did not experience chronic illness in their families compared to those who experienced chronic illness in their families, suggesting adverse selection. - Low enrollment among participants who complained about premium collection time compared to those who did not. |
| 18 | Ngowi and Nuru, 2023 | Manyara Region Tanzania | Enrollment | - Enrollment among participants with awareness about iCHF was significantly greater than that of their counterparts. - Participants who have a household member with chronic disease were more likely to enroll than those without a chronic disease member, suggesting adverse selection. - Participants with less income were more likely to enroll than those with higher income, contrary to popular beliefs. - Enrollment significantly higher among married women than their unmarried counterparts. - Enrollment odds significantly higher among larger household size than smaller ones |  |
| 19 | Nshakira-Rukundo *et al.*, 2019 | South-Western Uganda | Enrollment and renewal | - Households with higher access to information through radios, TV and newspapers had significantly higher odds of enrollment to CBHI as information helps enlighten them. - Trust on the programme increased enrollment. - Participants with positive perception about quality of services delivered were more likely to enroll than those with negative perception. - Wealth increase participation in CBHI program. - Employment in casual work increased odds of enrollment in CBHI. - Social capital and social cohesion increased enrollment in CBHI. - Belonging to catholic religion increased odds of enrollment in CBHI | - Belonging to a large burial group reduced enrollment. |
| 20 | Taddesse *et al.*, 2020 | Northwest Ethiopia | Enrollment | - Perception of affordability of premiums increased enrollment. - Participants with a history of illness in their households have high odds of enrollment to CBHI program, an adverse selection. - Being married increased odds of enrollment to CBHI. - Larger household size (4-6) has higher odds of enrollment to CBHI compared to smaller ones. |  |
| 21 | Kamau, N and Njiru, H | Rural Kenya | Enrollment | - Respondents ready to join if premiums were lowered below $23 from $37. - Subsidies to the CBHI scheme enabled the scheme to continue running, premiums were just 10%. | - Poor understanding of the concept of health insurance where non-members got discouraged from enrollment for the fact that there is no refund of fees if not utilized. - Lack of trust on scheme management by current and former members, who view management team as insensitive to client financial situation. - Non-members did not see incentive to enroll since members did not receive their preferred treatment. - Most residents believe in herbal medicine and transact by barter trade. - Lack of essential clinical services such as X-ray discouraged enrollment. - Perception that cost of premium is too high despite option to pay in instalments, discouraged enrollment. - Long distance to health facility discourages participants from seeking care. |
| 22 | Kamuzora, K. and Gilson, L. | Tanzania | Enrollment |  | - Communities fail to see the rationale of protecting against health risks. - Introduction of scheme policy at central level was not well received at the district level. - Policy bureaucracy limited the actions of the district managers which affected the workflow of the CBHI scheme. - Inability to pay annual premiums prevented enrollment. - Staff shortages prevented the wealthy participants from enrollment. - Insufficient supervision of health staff and other staff related issues resulted in low enrollment. - Poor quality of services discouraged enrollment. - Lack of comprehensive services and referral system discouraged enrollment. - Lack of choice for facility to use discouraged enrollment. |
| 23 | Desalegn *et al.*, 2023 | Ethiopia | Enrollment | - Having formal (primary education) show a positive association with CBHI enrollment. | - Poor knowledge about CBHI reduced the likelihood of enrollment by 52%. - Perception of disrespectful care by health workers decreased enrollment likelihood by 56%. - Unavailability of continuous laboratory services was significantly associated with lower CBHI enrollment. - Household heads perceiving premium collection time as inappropriate were less likely to enroll. - Households in the medium wealth category were less likely to enroll, possibly due to ability to pay out-of-pocket. |
| 24 | Elmi, Oladeji and Tahir, 2025 | Ethiopia | Enrollment | - Households with information about CBHI were more likely to enroll than those without. - Membership in a solidarity group significantly increased the likelihood of CBHI enrollment. - Households with higher annual income were more likely to be enrolled in the scheme. |  |
| 25 | Kassie *et al.*, 2025 | Ethiopia | Enrollment | - Women living in urban areas were more likely to enroll in CBHI than those in rural areas. - Women whose husbands had primary education were more likely to enroll than those whose husbands were uneducated. | - Wealthier women were less likely to enroll in community-based health insurance. - Household heads aged 40 and above were less likely to enroll compared to younger ones. - Women without media access were less likely to enroll than those with access. - Women from households with more than five family members were less likely to enroll in CBHI. - Women with two or fewer children under five were less likely to enroll compared to those with more. |
| 26 | Handebo *et al.*, 2023 | Ethiopia | Enrollment | - Women in Afar, Amhara, Oromia, Addis Ababa, and Dire Dawa had higher CBHI enrollment than those in Tigray. - Women with higher education levels were more likely to enroll in CBHI than non-educated women. - Women affiliated with Muslim, Catholic, traditional, or other religions were more likely to enroll than those affiliated with the Orthodox religion. | - Women aged 20–34 had lower odds of enrolling in CBHI than those aged 15–19. - Women living in rural areas were less likely to enroll than those in urban areas. - Protestant women were 28% less likely to enroll than Orthodox-affiliated women. - Women with larger family sizes were less likely to enroll in CBHI. - Female-headed households were 19% less likely to enroll in CBHI. - Women with more living children were less likely to enroll in CBHI. |
| 27 | Demsash, 2024 | Ethiopia | Enrollment | - Household heads with secondary education significantly increased CBHI enrollment, rising from 34.7% to 80.4%. - Households exposed to media were 1.4 times more likely to enroll in CBHI. - Household heads older than 35 years were 2.5 times more likely to enroll than those aged 15–24. | - Poor households were significantly less likely to enroll in CBHIS, with enrollment dropping from 45.2% to 5.6% as wealth decreased. - Increased media exposure was associated with a decreased likelihood of CBHI enrollment. - Households in Afar, Gambela, Harari, and Dire Dawa were significantly less likely to enroll in CBHIS. |
| 28 | Wodessa *et al.*, 2025 | Ethiopia | Enrollment | - Respondents with no formal education had higher odds of enrolling in CBHI than those with above-secondary education. - Participation in local solidarity practices (Equb or Edir) increased the likelihood of CBHI enrollment. - Respondents who perceived the quality of CBHI schemes as good were more likely to enroll in the scheme. - Households that had trust in the CBHI scheme were more likely to enrol. - Households that received essential drug provision were more likely to enroll in CBHI. - Respondents satisfied with CBHI services had higher odds of enrolling. | - Household heads with a negative perception of the CBHI scheme were 93.3% less likely to enroll. - Households with poor or middle wealth status were less likely to enroll than those with the richest status. |
| 29 | Debessa, Negeri and Dangisso, 2025 | Ethiopia | Enrollment | - Older individuals had a higher likelihood of enrolling in CBHI. - Larger family size increased the likelihood of CBHI enrollment. - Women from middle wealth index households were more likely to enroll than those from the poorest households. - Communities with higher levels of women’s autonomy showed greater CBHI enrollment. - Communities with higher literacy rates or formal education attendance had higher CBHI enrollment. |  |
| 30 | Kebede, 2024) | Ethiopia | Enrollment | - Shorter distance to the nearest health facility increased likelihood of CBHI enrollment. - Families with members holding official local government or cultural positions were more likely to enroll. - Trust in local CBHI scheme management positively influenced enrollment. | - Membership in a rotating saving and credit association reduced the likelihood of CBHI enrollment. |
